# Label-Free Acoustic Wave Detection of Myelin Basic Protein for Monitoring of Neurodegenerative Disease

**DOI:** 10.3390/bios16100564

**Published:** 2026-10-08

**Authors:** Sandro Spagnolo, Sára Mikuličová, Katharina Davoudian, Kiran Sontakke, Brian De La Franier, Michael Thompson, Tibor Hianik

**Affiliations:** 1 Faculty of Mathematics, Physics and Informatics, Comenius University, Mlynská Dolina F1, 84248 Bratislava, Slovakia; spagnolo2@uniba.sk (S.S.); sara.mikulicova@fmph.uniba.sk (S.M.); kiran.sontakke@fmph.uniba.sk (K.S.); tibor.hianik@fmph.uniba.sk (T.H.); 2 Department of Chemistry, University of Toronto, 80 St. George Street, Toronto, ON M5S 3H6, Canada; k.davoudian@mail.utoronto.ca (K.D.); brian.delafranier@mail.utoronto.ca (B.D.L.F.)

**Keywords:** myelin basic protein, acoustic wave biosensor, DNA aptamers, neurodegenerative disease, multiple sclerosis

## Abstract

Myelin basic protein (MBP) is released into cerebrospinal fluid during demyelination and is therefore a promising biomarker for the detection of neurodegenerative diseases such as multiple sclerosis. For the label-free detection of MBP, we developed an antifouling aptasensor based on thickness shear mode sensing. DNA aptamers were immobilized on gold-coated piezoelectric crystals functionalized with an engineered antifouling self-assembled monolayer based on 3-(2-mercaptoethoxy)propanoic acid (HS-MEG-COOH) combined with either 2-(2-mercaptoethoxy)ethanol (HS-MEG-OH) or dithiothreitol (DTT) as spacers. The binding of recombinant human MBP to the aptamer was confirmed with a corresponding decrease in resonance frequency in both phosphate-buffered saline (PBS) and artificial cerebrospinal fluid (aCSF). The aptasensor achieved a limit of detection of 11 ng mL^−1^ in PBS and 2 ng mL^−1^ in aCSF. PBS measurements were primarily applied to determine the aptasensor functionality, whereas experiments in aCSF evaluated MBP detection at concentrations relevant to reported levels in biological CSF. Selectivity was assessed with aptamer-free surfaces, cytochrome c, and human serum albumin (HSA) with much lower responses, while concentration-dependent MBP detection was maintained in aCSF despite the increased complexity of the matrix. The antifouling performance of the modified sensing surface was further demonstrated in aCSF by the significantly reduced frequency and dissipation responses compared to bare gold. With future validation in human cerebrospinal fluid (CSF) samples, the developed biosensor has the potential to enable sensitive, label-free detection of MBP in CSF for the identification and monitoring of neurodegenerative diseases.

## 1. Introduction

The myelin sheath is a multilamellar proteolipid membrane that is critical for neuronal insulation and the rapid conduction of nerve impulses, which is essential for cognition, memory, sensation, and motor functions [1]. An important structural component of the myelin sheath is myelin basic protein (MBP), an intrinsically disordered protein rich in arginine and lysine residues that does not possess a defined globular structure, allowing it to interact with various molecules and perform different functions [2]. MBP exists in several isoforms (14–21.5 kDa) with an isoelectric point around 11 [1] and is referred to as the executive protein due to its critical function in the formation of compact myelin sheath in the central nervous system (CNS) [3].

The abundance of MBP differs between the CNS and the peripheral nervous system (PNS). In the CNS, MBP is the second-most abundant myelin protein, with a dry mass of 30% of the total myelin protein mass. In contrast, MBP accounts for 5–18% of PNS myelin [3]. In human myelin, the 18.5 kDa isoform is the most abundant MBP isoform in the CNS that regulates myelin formation, compaction, and maintenance [2,4], while the 17.2 kDa isoform is the predominant MBP isoform in the PNS [3]. Besides isoform variations, MBP also experiences post-translational changes, such as phosphorylation, methylation, deamidation, acetylation of lysine, and citrullination of arginyl residues [2]. These post-translational modifications produce charged isomers C1–C8 of the 18.5 kDa isoform that affect its structural dynamics, impacting the ability to maintain a compact myelin sheath. C1 is the least modified and mostly cationic isomer, with a net positive charge of +19 at pH 7 [2]. The most extensively modified C8 isomer (due to citrullination of six arginine residues) has the lowest net positive charge of +13 at pH 7 [3]. Citrullinated MBP (citMBP) also plays a pathological role in the CNS, since citrullination causes incomplete protein-lipid interactions that disorganize the myelin sheath and make the membrane more vulnerable to rapid degradation by proteolytic enzymes. A proportion of 20% of MBP is citrullinated in healthy brains, while citMBP levels increase to 45% in the brains of patients with multiple sclerosis (MS), an autoimmune disease of the CNS involving damage to the myelin sheath [2]. In Marburg MS, an aggressive type of disease, citMBP increases to 90% of total MBP [5].

Cerebrospinal fluid (CSF) can be considered as a liquid biopsy that reflects the health of the nervous system. The CSF is a fluid mixture from the choroid plexus and interstitial fluid originating from the brain, spinal cord, meninges, and blood vessels, with a total volume of about 125–150 mL in the average adult. This volume is turned over about four times each day, and approximately 600 mL of CSF is generated on a daily basis. Biomarkers in the CSF can be associated with neurodegenerative diseases, such as tau and neurofilament proteins as markers of axonal damage, glial fibrillary acid protein for gliosis, and MBP for demyelination [6]. Demyelination causes impaired neuronal conduction that can present as various symptoms characterized by increased levels of MBP in CSF, typically exceeding 6.2 ng mL^−1^ in the most common demyelinating disease, MS. Other demyelinating diseases include acute disseminated encephalomyelitis (ADEM), Guillain–Barré syndrome (GBS), and Charcot–Marie–Tooth disease (CMT) [1]. During polysymptomatic exacerbations in MS, MBP concentrations can reach 22.3 ng mL^−1^ in CSF. Current diagnosis of MS primarily relies on magnetic resonance imaging and immunological testing that are time-consuming, costly, and require specialized facilities. Clinically, MBP is typically measured in CSF samples using enzyme-linked immunosorbent assays (ELISAs) [7]. Although ELISA is a sensitive technique, it depends on time-consuming incubation steps and specialized laboratory infrastructure. Therefore, rapid and sensitive detection strategies with biosensing could complement established laboratory approaches and enable earlier assessment of demyelinating diseases.

Biosensors are promising tools for effective MBP detection by interfacing a bioreceptor selective for MBP with a transducer. Among bioreceptors, DNA aptamers offer several advantages, including high affinity, excellent stability, and straightforward synthesis and surface immobilization [8]. Aptamers are short single-stranded oligonucleotides that specifically bind to a target based on their three-dimensional conformation that depends on external conditions such as pH and ionic strength [9]. The specific nucleotide sequence is selected through the process of Systematic Evolution of Ligands by Exponential Enrichment (SELEX), which identifies the aptamer sequence with high affinity toward a target molecule [10].

Several DNA aptamers targeting MBP have been developed and studied for therapeutic purposes. For example, MBPcl3 and MBPcl9 sequences against mouse MBP showed limited specificity [11]. SELEX for crude murine myelin produced the G-quadruplex-forming aptamer LJM-3064 [12] and its derivative, LJM-5708, demonstrated higher affinity for binding MBP [13]. Although LJM-5708 was originally selected against mouse MBP, its binding to human oligodendroglioma cells has also been reported, providing evidence of cross-species recognition [13]. The first biosensor for MBP detection used the LJM-5708 aptamer, where electrochemical impedance spectroscopy (EIS) was applied to detect MBP in buffer and artificial cerebrospinal fluid (aCSF). Limits of detection of 0.01 ng mL^−1^ [14] and 0.65 ng mL^−1^ [15] were obtained for mouse and human MBP, respectively, with the latter providing further evidence of the applicability of LJM-5708 to human MBP detection.

In this work, a thickness shear mode sensor with dissipation monitoring (TSM-D) was applied for the label-free and real-time acoustic wave detection of MBP using an engineered antifouling aptamer interface for the first time. The biomolecular interaction of MBP-aptamer binding is monitored through changes in resonance frequency (Δƒ) and energy dissipation (Δ*D*) at the surface of the resonating piezoelectric crystal. A decrease in the resonance frequency is typically associated with the adsorption of biomolecules, while dissipation is due to the mechanical properties of the adsorbed layer. The adsorption of thin, rigid adlayers produces frequency shifts that are directly proportional to the mass adsorbed, based on the Sauerbrey equation [16]:

$$\Delta ƒ=-\frac{2n{ƒ}_{0}^{2}}{A\sqrt{\rho_{q}\mu_{q}}}\Delta m$$

where Δƒ is the resonance frequency shift; *n* is the overtone number; ${ƒ}_{0}$ is the fundamental frequency; *A* is the active crystal area; Δ*m* is the adsorbed mass; and $\rho_{q}$ and $\mu_{q}$ are the density and shear modulus of quartz, respectively. However, the adsorption of viscoelastic films also causes changes in energy dissipation, making the Sauerbrey equation no longer applicable; therefore, viscoelastic models must be applied to estimate the quantity of mass adsorbed.

The level of rigidity of an adsorbed layer can be estimated from the ratio between dissipation and frequency changes according to the following relationship [17]:

$$\frac{\Delta D_{n}}{\Delta {ƒ}_{n}}\ll \frac{1}{{ƒ}_{0}}$$

Studies have shown that layers with $\frac{\Delta D}{\Delta f}\;<\;0.1\times 10^{-7}$ Hz^−1^ largely behave as rigid films, while larger values of $\frac{\Delta D_{n}}{\Delta f_{n}}>4\times 10^{-7}Hz^{-1}$ indicate an increase in viscoelastic contributions. This estimation therefore determines whether the Sauerbrey or viscoelastic models can be applied for the reliable estimation of adsorbed mass on the surface of the piezoelectric crystal. Previous studies have shown that adlayers with ΔD/Δf < 10^−8^ Hz^−1^ behave predominantly as rigid films, allowing for reliable estimation of the surface mass density using the Sauerbrey equation, whereas substantially larger values indicate increasing viscoelastic contributions [18].

The biosensor combines a mixed antifouling thiol-based self-assembled monolayer with the LJM-5708 DNA aptamer and acoustic TSM-D transduction for MBP detection in aCSF. The antifouling sensing surface was designed to minimize nonspecific adsorption while maintaining aptamer accessibility for MBP recognition, enabling measurements in complex biological matrices [19]. This surface strategy does not require protein-based blocking agents and therefore avoids an additional adsorbed layer. Such blocking agents could contribute to the mass coupling to the sensor surface and introduce viscoelastic effects on the acoustic response [20], potentially complicating the interpretation of analyte-binding measurements. Previous MBP aptasensors that used LJM-5708 have relied on electrochemical transduction [14,15]. On the other hand, this study applied an acoustic TSM-D sensor for label-free and real-time monitoring of recombinant human MBP. To the best of our knowledge, an acoustic wave LJM-5708 aptasensor has not previously been reported for MBP detection. A distinct advantage of TSM-D is the continuous monitoring of the sensing interface under flow, allowing both aptamer immobilization and MBP binding to be followed in real time [21]. Simultaneous monitoring of frequency and dissipation further provides information on the properties of the interfacial layer (e.g., surface associated mass and aptamer surface coverage) and allows for assessment of the applicability of the Sauerbrey relationship. The study therefore combines acoustic detection, real-time monitoring of sensor functionalization and target binding, and interfacial characterization within a single sensing platform.

## 2. Materials and Methods

### 2.1. Materials

All standard chemicals were purchased from Merck (Darmstadt, Germany). For buffers and solutions, deionized ultrapure water (DI) was used (Purelab Classic-UV ELGA, High Wycombe, UK). A denaturing solubilization buffer consisting of 6 M urea, 50 mM Tris-HCl, 100 mM NaCl, and 1% (v/v) Triton X-100 at pH 8.0 was used for protein solubilization after its expression. Standard phosphate-buffered saline (PBS) was used (10 mM, pH 7.4 containing 10 mM Na_2_HPO_4_, 10 mM NaH_2_PO_4_, 137 mM NaCl and 2.7 mM KCl) as the running buffer for acoustic measurements unless otherwise specified. His-binding buffer consisted of 300 mM NaCl, 20 mM Tris, 20 mM imidazole, and 1 mM CaCl_2_, pH 7.2. The HEPES buffer consisted of 20 mM HEPES, 1 mM CaCl_2_, pH 7.4. All buffers were filtered through a 0.2 µm filter (Merck-Millipore, Darmstadt, Germany).

Material for MBP production: BenchMark™ Pre-stained Protein Ladder, protease inhibitor cocktail (BioShop, Burlington, ON, Canada), and LB powder (Invitrogen, Thermo Fisher Scientific, Waltham, MA, USA) were obtained from MedStore (Toronto, ON, Canada). DNA aptamer specific to MBP labeled as LJM-5708 developed by Wilbanks et al. [13] with the sequence 5’—GGG TTA GCG GGT GAG GTG GGT TTT TTT TTT—C7 Amino- 3’ was obtained from Genecust (Boynes, France). The aptamer was dissolved in DNase-free water to a stock concentration of 0.1 mM and stored in aliquots at −20 °C. Prior to every measurement, an aptamer aliquot was defrosted and diluted to a working concentration of 1 µM in PBS.

For immobilization of the amino-terminated aptamer, the gold-coated sensing surfaces were modified with a 0.5 mM mixed self-assembled monolayer of (HS-Mix), consisting of 0.25 mM 3-(2-mercaptoethanoxy) propanoic acid (HS-MEG-COOH) with either 0.25 mM 2-(2-mercaptoethoxy)-ethanol (HS-MEG-OH) or dithiothreitol (DTT) [22]. HS-MEG-OH and HS-MEG-COOH were synthesized at the University of Toronto (Canada) based on a reported protocol [22]. The carboxyl groups of HS-MEG-COOH were activated with 20 mM N-hydroxysuccinimide (NHS) and 50 mM N-(3-dimethylaminopropyl)-N’-ethyl carbodiimide hydrochloride (EDC) in MES buffer (pH 5.5), enabling covalent coupling of the amino-modified aptamer. Remaining activated carboxylic acid groups without bound aptamer were blocked with 0.1 M diethanolamine (diETA) to minimize nonspecific adsorption.

### 2.2. Recombinant Myelin Basic Protein

#### 2.2.1. Plasmid Design and Transformation of Escherichia coli

Recombinant MBP was designed with pRSET_B as the expression vector. The coding sequence was cloned into the multiple cloning site downstream of the T7 promoter and in frame with the vector-encoded N-terminal 6×His tag and Xpress™ epitope for affinity purification and immunodetection. The MBP gene was inserted into the multiple cloning site of the vector to produce a recombinant plasmid of 3443 bp. The pRSET_B vector also includes the bla gene to provide ampicillin resistance for plasmid selection. The recombinant plasmid was applied for the heterologous expression of His-tagged MBP in competent *Escherichia coli* (*E. coli*) BL21 (DE3) cells. The amino acid sequence of recombinant MBP and a schematic representation of the recombinant pRSET_B expression plasmid is provided in the Supplementary Materials, Figure S1.

Competent *E. coli* BL21 cells were transformed with the pRSET_B expression plasmid encoding recombinant human myelin basic protein (MBP) carrying a 6×His tag, following a modified protocol reported by De La Franier and Thompson [23]. Transformed cells were grown on an agar plate with ampicillin (100 mg L^−1^) overnight at 37 °C to select for cells containing the plasmid, then stored at 4 °C. A single colony was cultured into LB broth with ampicillin (100 mg L^−1^), overnight at 37 °C, and cell stocks were made from this solution and frozen (30% glycerol).

#### 2.2.2. Recombinant Expression of MBP

A starter culture was produced by inoculating either a transformed *E. coli* BL21 (DE3) colony from a stock agar plate or 1 mL of a stored cell stock into 40 mL of lysogeny broth (LB) with 100 mg L^−1^ ampicillin. The culture was incubated at 37 °C overnight on an orbital shaker. The culture was then used to inoculate 2 L of LB with 100 mg L^−1^ ampicillin. The cells were grown at 37 °C until an optical density at 600 nm (OD_600_) of 0.4–0.8 was reached. Recombinant protein expression was induced with isopropyl β-D-1-thiogalactopyranoside (IPTG) to a final concentration of 0.2 mM. The culture was incubated overnight at room temperature on an orbital shaker, and the cells were harvested following 45 min of centrifugation at 2000 rpm.

#### 2.2.3. Purification of Recombinant MBP

The bacterial cell pellet was resuspended in lysis buffer that contained 1 mg mL^−1^ lysozyme, 40 µL of protease inhibitor cocktail, 5% glycerol, 0.1% Triton-X, 20 mM imidazole, and 20 mM Tris pH 7.2. The cells were sonicated for 30 min, before DNase 1 was added and the solution was incubated at 37 °C on an orbital shaker for 30 min. The lysate was centrifuged at 14,500 rpm for 45 min. The pellet was resuspended in a denaturing solubilization buffer that contained 6 M urea (approximately 5 mL of denaturing buffer per gram of pellet) and placed on an orbital shaker for 1 h at ambient temperature. The suspension was then centrifuged at 15,000 rpm for 20 min and the supernatant was repeatedly buffer exchanged into His-binding buffer with 10 kDa molecular weight cut-off centrifugal ultrafiltration devices (Amicon Ultra, Millipore Sigma, Darmstadt, Germany).

Recombinant MBP was purified with Ni-NTA agarose resin affinity chromatography that was equilibrated with His-Binding buffer (20 mM Tris, 300 mM NaCl, 5 mM Imidazole, 1 mM CaCl_2_, pH 7.6). Following incubation of the protein solution with the agarose resin for 1 h at 37 °C under gentle mixing, the resin was transferred to a chromatography column. Unbound proteins were collected as column flow-through (CFT), which was followed by washing with 15 column volumes of His-binding buffer. Bound recombinant MBP was then eluted with His-binding buffer that contained a higher concentration of imidazole (220 mM, 4 mL).

The purified protein was then buffer-exchanged into HEPES buffer (20 mM HEPES, 1 mM CaCl_2_, pH 7.4) and concentrated with the previously described 10 kDa centrifugal filters (Amicon Ultra, Millipore Sigma). The purification of the protein was evaluated with 15% SDS polyacrylamide gel electrophoresis (SDS-PAGE) to analyze the CFT, washes, and elution fractions. The molecular weight of the purified protein was confirmed with SDS-PAGE. The protein size was characterized with a Zeta Sizer Nano (Malvern, UK), while concentration was calculated using a UV1700 UV-VIS spectrometer (Shimadzu, Shanghai, China). The presence of recombinant His-tagged MBP was further confirmed with acoustic wave sensing by its binding to Ni-NTA-functionalized TSM disc surfaces.

### 2.3. Artificial Cerebrospinal Fluid

Samples of aCSF were used to mimic the composition of CSF. CSF is a liquid that has a high percentage of ions, but a low percentage of proteins, with albumin representing one of its major protein components. The composition of aCSF was chosen based on the literature and adapted for acoustic wave measurements [6,24,25,26]. The aCSF was freshly prepared directly before measurements to prevent precipitation during long-term storage from three 10× concentrated stock solutions (A, B and C). Solution A consisted of NaCl, KCl and NaH_2_PO_4_∙H_2_O. Solution B contained the divalent MgCl_2_ and CaCl_2_ ions with D-glucose and urea. Human serum albumin (HSA) was stored separately as solution C. The final composition of the aCSF was 137 mM NaCl, 2.5 mM KCl, 1.25 mM NaH_2_PO_4_, 1 mM MgCl_2_, 2 mM CaCl_2_, 0.65 mM urea, 4 mM D-glucose and 6.15 µM human serum albumin.

### 2.4. Acoustic Wave Sensing

Acoustic wave measurements were performed using AT-cut quartz crystals (8 MHz fundamental frequency) with gold electrodes (0.2 cm^2^ area deposited on each side) purchased from Total Frequency Control Ltd. (Storrington, UK). Measurements were performed with a Sark-110 vector analyzer (Seed, Shenzhen, China) that recorded the fundamental frequency and higher harmonics (n = 1, 3, 5, 7) collected with Python (3.13.6) software. The quartz crystals were mounted in an acrylic flow-through cell LSC01 with a volume of 100 μL [27] (Johannes Kepler University, Linz, Austria) connected to a Genie Touch syringe pump (Kent Scientific, Torrington, CT, USA). Measurements were conducted at room temperature with solutions flowing at a rate of 50 µL min^−1^. PBS was used as the running buffer between samples. The obtained data were fitted with a Python code based on a reported model [28]. All results were then processed with GraphPad Prism 10 and OriginPro 7.5 SR6.

The crystals were cleaned using basic piranha solution (1:1:3 v/v 28–30% NH_4_OH, 30% H_2_O_2_, Milli-Q water (Merck, Kirkland, QC, Canada) at 75 °C) with three 25 min cycles and with Milli-Q water rinses in between. After cleaning, the crystals were rinsed with Milli-Q water and stored in absolute ethanol.

Functionalization of the crystals involved incubation overnight in 0.5 mM HS-MEG-Mix at room temperature, followed by an ethanol rinse and drying with a gentle flow of nitrogen. Subsequent surface functionalization steps were performed in flow. The carboxylic acid groups of HS-MEG-COOH were activated with 20 mM NHS and 50 mM EDC in MES for 45 min. To confirm the presence of a histidine tag on recombinant MBP, the NHS-activated HS-MEG-COOH crystals were exposed to Ni-NTA in PBS (2 mg mL^−1^ N(α), N(α) bis-(carboxymethyl)-L-lysine disodium salt monohydrate (ab-NTA) and 2 mg mL^−1^ NiCl_2_) for 1 h, producing a MEG-NTA functionalized surface. The binding of recombinant MBP through Ni-NTA/His-tag interactions was evaluated in real time with the TSM sensor: following frequency stabilization in PBS, recombinant MBP was injected for 1 h, and PBS was re-introduced and the frequency shift was evaluated.

For the detection of recombinant MBP, NHS-activated HS-MEG-COOH-modified crystals were incubated with amino-terminated aptamer for 4.5 h. To prevent non-specific adsorption, 0.1 M diethanolamine was incubated for 45 min. MBP solutions (ranging from 0.005 to 10 μg mL^−1^) in PBS or aCSF were flowed for 60 or 20 min, respectively. Triplicate measurements were performed for each concentration.

## 3. Results and Discussion

### 3.1. Characterization of Recombinant Myelin Basic Protein

#### 3.1.1. SDS-PAGE Analysis

Characterization of the purification fractions by SDS-PAGE (Supplementary Materials, Figure S2) showed that the column flow-through fraction (CFT) contained numerous proteins that did not bind with the Ni-NTA affinity column (lane B). The elution fraction (lane C) showed a predominant band at approximately 21 kDa, which is consistent with the expected molecular weight of recombinant His-tagged MBP and in agreement with the dynamic light scattering results described in Section 3.1.3. The His-binding buffer washes also exhibited faint protein bands while lacking the band at 21 kDa (lanes D–F), suggesting that most of the weakly bound contaminants were efficiently removed during the CFT and washing steps while recombinant MBP remained bound in the column before elution. Although negligible residual impurities seem to be present, the SDS-PAGE gel indicates that recombinant MBP was successfully expressed and purified.

#### 3.1.2. UV-Vis Characterization

To determine the concentration of recombinant MBP, its absorbance was measured at 276 nm (Supplementary Materials, Figure S3). The Beer-Lambert law was applied:

$$A_{276}=\varepsilon_{276}\cdot c\cdot l$$

where $A_{276}$ is the measured absorbance at 276 nm, $\varepsilon_{276}$ is the molar extinction coefficient, *c* is the concentration, and *l* is the optical path length (1 cm). The theoretical molar extinction coefficient was determined from the amino acid sequence based on the contributions of aromatic residues and disulfide bonds [29]:

$$\varepsilon_{276}=5000\cdot n_{Try}+1490\cdot n_{Tyr}+125\cdot n_{Cystine}$$

where $n_{Try}$, $n_{Tyr}$ and $n_{Cystine}$ represent the number of tryptophan, tyrosine, and cystine (disulfide bonds) residues, respectively. The calculation involves the N-terminal Xpress epitope and a hexahistidine tag encoded by the pRSET_B expression vector. For the studied recombinant MBP, which contained two tryptophan residues, six tyrosine residues (which includes one contributed by the Xpress tag), and no disulfide bonds, the theoretical molar extinction coefficient is 19,940 M^−1^ cm^−1^. This value is consistent with an experimentally reported extinction coefficient for a similar recombinant MBP isoform [30].

The UV-Vis spectrum of purified recombinant MBP had an absorbance of 0.149 at 276 nm (Figure S3), corresponding to an estimated concentration of 0.157 mg mL^−1^. This represents the theoretical concentration of recombinant MBP, assuming that the protein is largely monodispersed and present in monomeric form. To further determine the state of recombinant MBP and assess potential aggregates, protein solutions were assessed with dynamic light scattering.

#### 3.1.3. DLS and Aggregation Assessment

The hydrodynamic properties of purified recombinant MBP were characterized with dynamic light scattering. Figure S4A shows a number distribution with a main protein population exhibiting a hydrodynamic diameter of 9.12 ± 1.46 nm, confirming successful purification. Similarly, the volume distribution in Figure S4C has a main population around 9.53 ± 1.54 nm, which accounts for 99.3% of the total particle volume. These results demonstrate a predominantly monodisperse solution of recombinant MBP, confirming that the protein was successfully purified. The observed hydrodynamic diameter indicates that MBP is present in a predominantly non-aggregated state, which as an intrinsically disordered protein, likely adopts a more extended conformation in solution than compact globular proteins of similar molecular weight [31].

Although the intensity distribution of Figure S4B indicates three distinct protein populations around 9.92 ± 1.20 nm (10.6%), 60.85 ± 6.68 nm (8.9%), and 524.6 ± 67.42 nm (80.5%), the volume distribution (Figure S4C) shows that the majority of the sample (99.3%) is made up of MBP with diameters around 9.53 ± 1.54 nm. In contrast, the larger particles make up only 0.4% and 0.3% of the total sample volume. The discrepancies in the percent distribution of MBP in the intensity and volume distributions are characteristic of DLS, since the intensity of scattered light increases approximately with the sixth power of particle diameter. Therefore, trace amounts of possible large MBP aggregates can cause disproportionately strong scattering signals and dominate the intensity distribution despite representing only a negligible fraction of the total population. The results confirm that the purified protein samples predominantly contain non-aggregated recombinant MBP with negligible trace quantities of larger aggregates.

The above DLS measurements were performed at a protein concentration of 0.157 mg mL^−1^, which confirmed that the majority of MBP was present in a predominantly non-aggregated state. To investigate the concentration-dependent behaviour of recombinant MBP, the initial protein suspension was concentrated approximately tenfold by centrifugal spin filtration. The resulting concentrate was then subjected to two additional sequential twofold concentration steps using the same procedure, generating three progressively more concentrated samples, subsequently characterized with DLS to assess the behaviour of the protein and determine the concentration-dependent aggregation of recombinant MBP. However, since the main objective of this study was to express purified recombinant MBP and detect the protein in aCSF, neither the concentration threshold at which aggregation or precipitation occurs nor the kinetics and reversibility of aggregate formation were investigated.

The concentrated recombinant MBP samples showed a progressive increase in hydrodynamic diameter due to aggregation. The number distribution data of the least concentrated sample (Sample 1) had a main protein population diameter of 49.98 ± 17.55 nm (Figure S5A), while the intensity distribution showed major populations about 276.6 ± 301.8 nm and 3632 ± 1226 nm (Figure S5B). The second more concentrated sample (Sample 2) showed a further increase in size, with a diameter of 97.95 ± 23.29 nm in the number distribution (Figure S5C) and additional populations at 567.1 ± 158.6 nm and 693.1 ± 199.2 nm in the intensity distribution (Figure S5D). The most aggregation was present in Sample 3, which showed diameters of 175.1 ± 95.59 nm in the number distribution (Figure S5E) and 381.5 ± 199.0 nm in the intensity distribution (Figure S5F). Overall, these results indicate that increasing the concentration of recombinant MBP in HEPES buffer above approximately 1 mg mL^−1^ promoted MBP aggregation, shifting the populations toward progressively larger aggregated species.

The concentration-dependent increase in the hydrodynamic diameter of recombinant MBP samples is consistent with the physicochemical properties of MBP. Due to its intrinsically disordered structure, high density of positively charged amino acid residues, and conformational flexibility, MBP typically aggregates in solution, particularly at elevated concentrations and with a pH near to the neutrality. These characteristics lead to the formation of oligomeric assemblies and larger aggregates of MBP [32,33]. The DLS measurements demonstrate that purified recombinant MBP also undergoes aggregation at higher concentrations, and the initial concentration maintains a predominantly non-aggregated state of MBP, which is suitable for aptasensor detection of the protein. For the subsequent TSM experiments, the purified recombinant MBP was diluted to a working concentration of 0.0856 mg mL^−1^, from which all serial dilutions were prepared.

Although the present experiments do not distinguish aptamer recognition of non-aggregated and aggregated MBP, the substantially lower MBP concentrations used for aptasensor measurements compared with the concentrations at which progressive aggregation was observed with DLS indicate that the sensing experiments were conducted under conditions expected to favour predominantly non-aggregated MBP.

#### 3.1.4. Confirmation of Recombinant MBP Binding

Ni-NTA-coated TSM discs were used to directly detect recombinant MBP, which contains a histidine tag. Measurements were performed at the third overtone. After addition of MBP to the disc, a rapid decrease in the measured frequency occurred by approximately 140 Hz (corresponding to a normalized frequency shift of approximately 47 Hz; Supplementary Materials, Figure S6). The subsequent PBS rinse resulted in a stabilized frequency that increased by only 20 Hz (6.7 Hz after normalization), producing a net frequency shift following MBP binding of approximately −120 Hz (corresponding to a normalized frequency shift of approximately −40 Hz). This suggests that MBP contains a His tag and binds to the Ni-NTA-functionalized surface, as most of the bound protein remained on the sensing surface after washing with PBS.

### 3.2. Detection of Myelin Basic Protein

**Figure 1:**
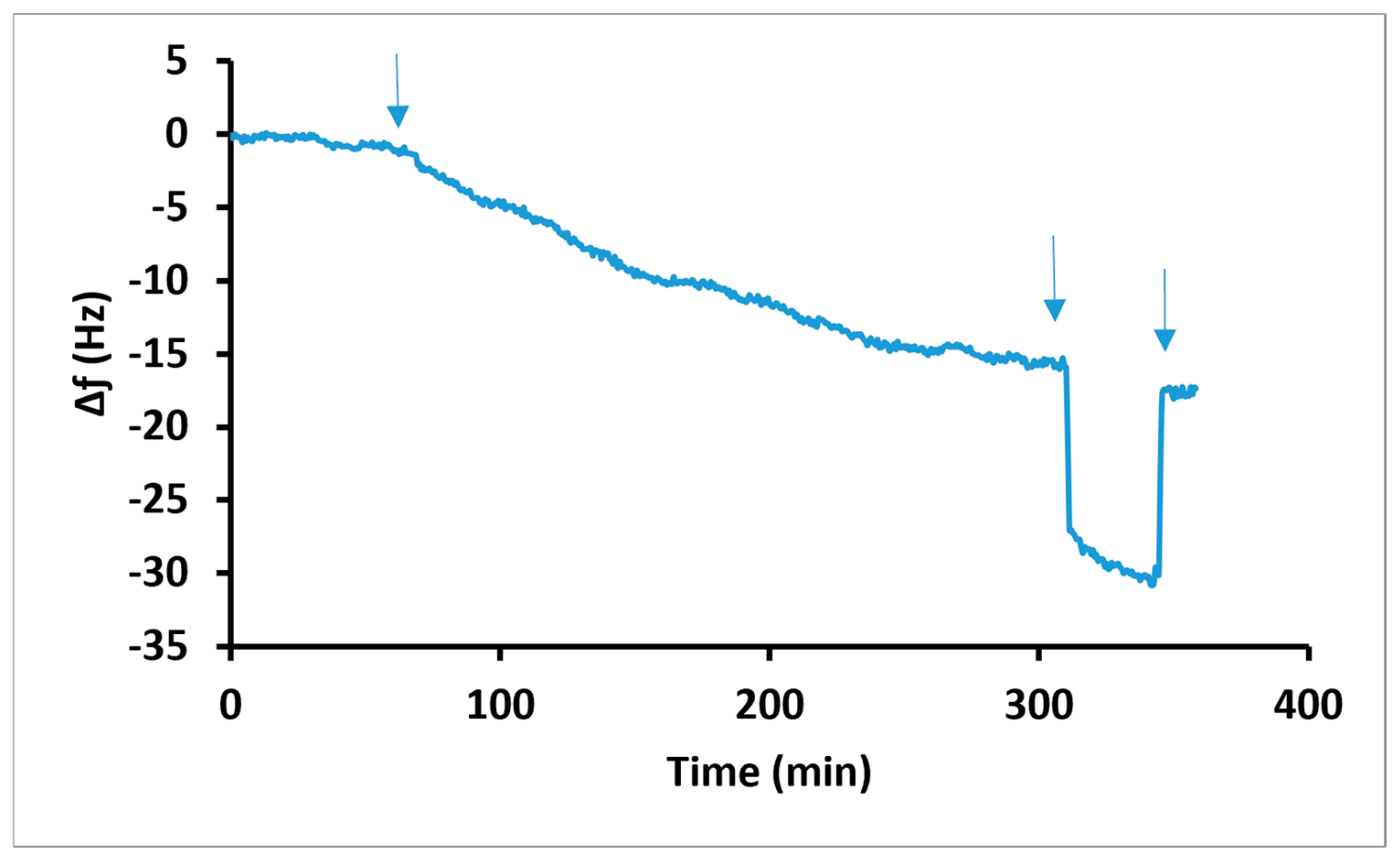
TSM sensor response of the frequency shifts during aptamer immobilization (first arrow) followed by diethanolamine blocking (second arrow) and buffer rinsing (third arrow).

Before MBP detection, the functionalization of the aptasensor was monitored by observing the normalized resonance frequency shifts during binding (Figure 1). Immobilization of the aptamer through NHS/EDC coupling produced a normalized frequency shift of −15.45 Hz, while the subsequent ethanolamine blocking of activated NHS-carboxyl groups produced a smaller frequency shift of −1.97 Hz.

**Figure 2:**
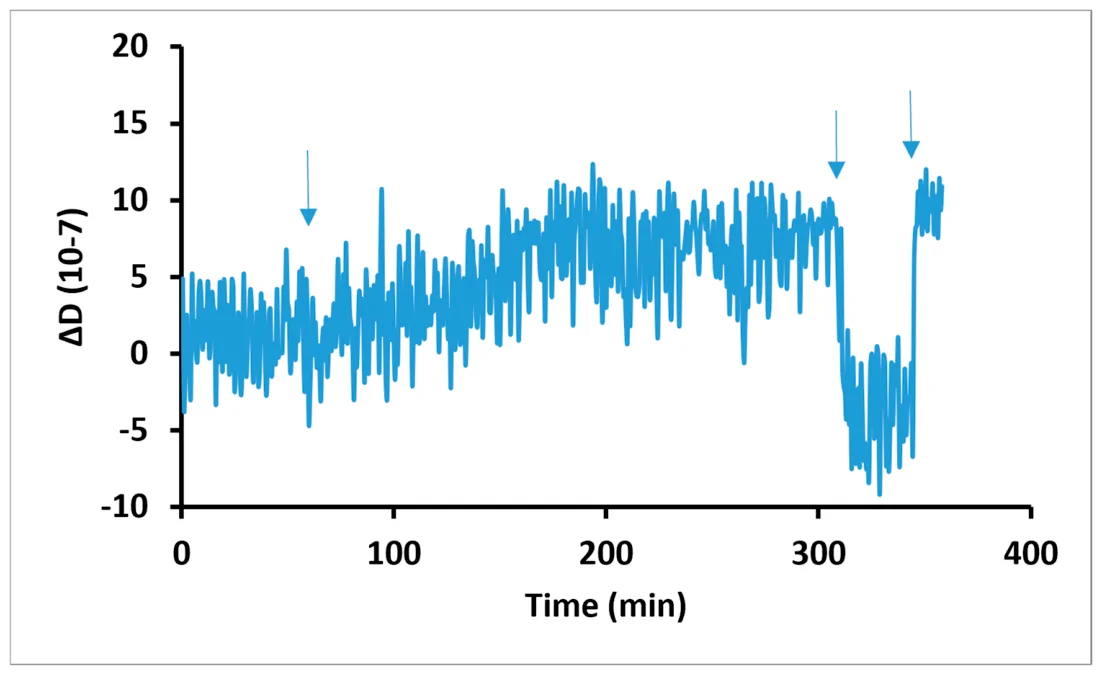
Real-time acoustic wave sensor response of the dissipation during aptamer immobilization (first arrow) and ethanolamine blocking (second arrow) and buffer rinsing (third arrow).

The change in energy dissipation was minimal throughout the functionalization of the aptamer and diethanolamine, indicating that the aptasensor layer is relatively thin and rigid (Figure 2). Only a slight increase in dissipation (Δ*D* = 6.33 × 10^−7^) was observed following aptamer immobilization, suggesting that the surface modification experienced minimal changes in viscoelastic properties.

The $\frac{\Delta D}{\Delta ƒ}$ ratio corresponding to the immobilization of aptamer was $0.4\;\times \;10^{-7}$ Hz^−1^, which is closer to the ratio for rigid films ($\frac{\Delta D_{n}}{\Delta f_{n}}<0.1\times 10^{-7}Hz^{-1}$) than viscoelastic films ($\frac{\Delta D_{n}}{\Delta f_{n}}>4\times 10^{-7}Hz^{-1}).$ This suggests that the aptamer layer does not behave like a completely elastic and rigid layer, but experiences some minimal viscoelasticity. However, the Sauerbrey equation can still be applied to approximate surface mass on the TSM crystal. Assuming an average molecular mass of 6.15 kDa for the LJM-5708 aptamer and an aptamer density of 1.7 g cm^−3^, a single aptamer has an approximate volume, radius, and molecular area of 6.0 nm^3^, 1.13 nm, and 4.0 nm^2^, respectively. Therefore, an ideally packed monolayer contains about 4.5–5.0 × 10^12^ aptamers on an active sensor area of 0.2 cm^2^. Such a monolayer would have a theoretical surface mass density of approximately 232–256 ng cm^−2^. In comparison, the experimentally determined surface mass density is approximately 106.6 ng cm^−2^, which corresponds to about 2.1 × 10^12^ aptamer molecules, representing 42–46% of the theoretical maximum coverage of aptamers on the active sensing area.

The theoretical calculation is based on the assumptions that aptamer molecules are compact and spherical with ideal packing, representing the upper limit of aptamers on the sensing area. However, the aptamer is expected to have a larger lateral shape due to its folded G-quadruplex structure, its terminal linker, and conformational flexibility upon surface attachment. Also, electrostatic repulsion between the negatively charged aptamer molecules, together with the hydration shell and counterions surrounding the aptamer molecules in an aqueous solution, is expected to reduce the packing density of the aptamer layer. Furthermore, the mass measured by TSM-D in liquids includes not only the biomolecular mass but also coupled water molecules and ions. As a result, the lower experimentally determined aptamer surface coverage is consistent with the steric, electrostatic, and hydration constraints that are associated with a hydrated and non-ideally packed aptamer layer.

The antifouling performance of the HS-MEG-COOH-based aptasensor was evaluated by comparing the TSM-D response of bare gold and the aptasensor upon exposure to aCSF. Compared with conventional alkylthiol SAMs based on 11-Mercaptoundecanoic Acid (MUA) and mercaptoalcohol spacers, MEG-based interfaces incorporate oligo(ethylene glycol) units that promote surface hydration and reduce nonspecific interactions, while the terminal carboxyl group of HS-MEG-COOH enables covalent probe immobilization. In our previous work, MEG-based SAMs showed improved antifouling behaviour compared with conventional MUA-based surfaces [22]. For the present aCSF experiments, DTT was used as a spacer in combination with HS-MEG-COOH, based on its lower dissipation response [22] and its successful application in antifouling sensing surfaces [34]. Bare gold exhibited a frequency shift of −63.45 Hz, whereas the aptasensor showed a significantly lower shift of −11.4 Hz, corresponding to an ~82% reduction in the frequency response. The dissipation change was also reduced from 8.28 × 10^−6^ for bare gold to 3.09 × 10^−6^ for the aptasensor (Figure S7). These results indicate that the functionalized sensing interface substantially reduces matrix-associated interactions compared with bare gold, although residual interactions with aCSF remain.

**Figure 3:**
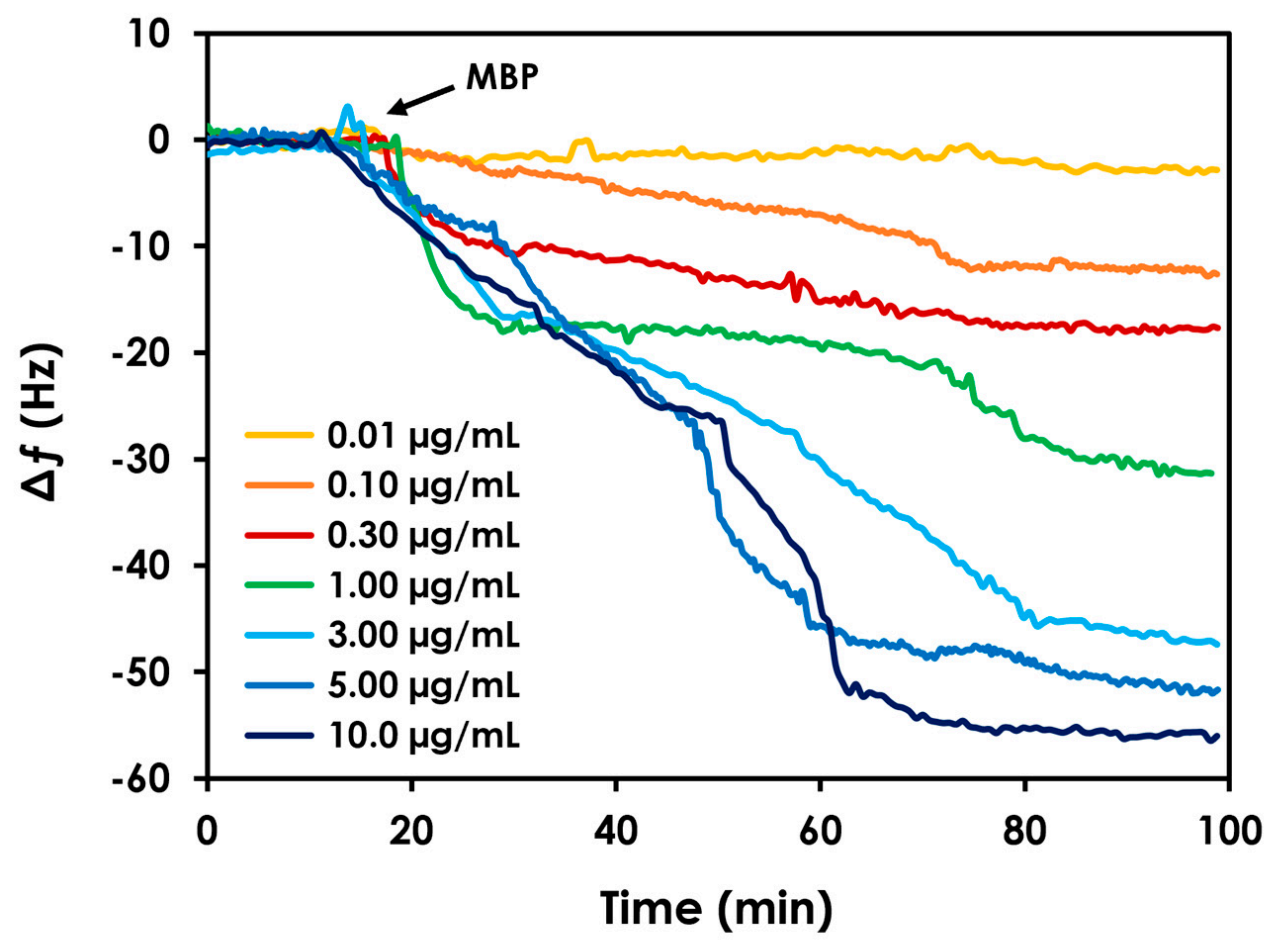
TSM sensor response to recombinant MBP in phosphate-buffered saline. Arrow indicates the addition of MBP to the sensing surface. The MBP concentrations are shown in the inset.

**Figure 4:**
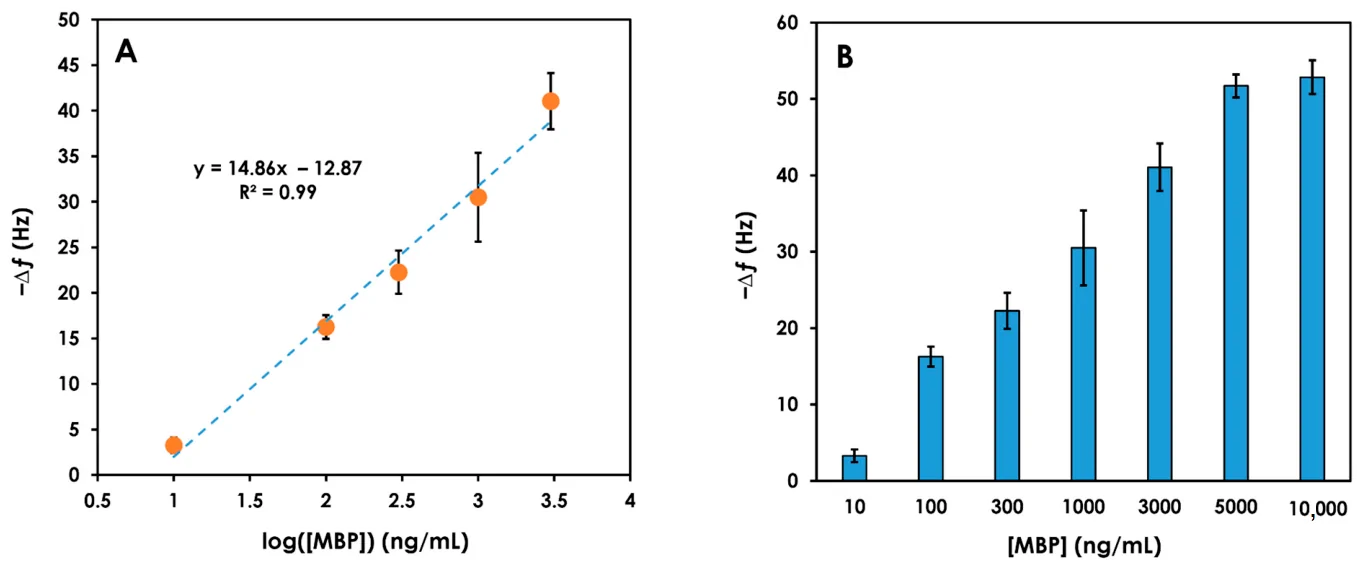
(**A**) The calibration curve plotting frequency shifts (∆ƒ) against the logarithm of recombinant MBP concentrations, described by the linear regression equation, y = 14.86x − 12.87 (R^2^ = 0.99), and (**B**) average responses of the acoustic wave aptasensor to recombinant MBP in PBS. The results are mean ± SD obtained from three independent experiments.

Solutions of MBP in PBS were prepared between 0.01 and 10 µg mL^−1^. Once a baseline was established under PBS flow, each MBP solution was flowed over an aptamer-modified disc. For these tests, the signal typically decreased slowly and reached a new stable baseline after approximately one hour (Figure 3). As the MBP concentration increased, the magnitude of the frequency shift increased from 3.3 Hz at 0.01 µg mL^−1^ to 51.7 Hz at 5 µg mL^−1^ (Table S1). Increasing the concentration to 10 µg mL^−1^ caused only a slight additional shift compared with 5 µg mL^−1^, suggesting the sensor approaches saturation at approximately 5 µg mL^−1^. The corresponding calibration curve and average sensor response for various concentrations of MBP in PBS is shown on the Figure 4.

The analytical performance of the sensor was initially evaluated in PBS to verify the functionality of the recognition strategy. A calibration curve was constructed using five different MBP concentrations corresponding to the linear region of the response. Higher concentrations resulted in deviations from linearity, likely due to progressive saturation of the sensing interface, and were therefore excluded from the LOD calculation. Since the purpose of the PBS experiments was primarily to demonstrate proof-of-concept operation, a more extensive concentration range was subsequently investigated in artificial cerebrospinal fluid (aCSF), providing a more complex matrix for evaluating sensor performance.

The limit of detection (LOD) was determined from the calibration plot performed in the linear region of the Δ*f* versus log_10_(C) plot. The LOD signal was first calculated as

$$y_{LOD}=3.3\cdot \sigma ,$$

where *σ* is the standard deviation of replicate measurements at the lowest concentration. For the lowest tested MBP concentration (10 ng mL^−1^), σ was 0.8 Hz, producing an LOD signal of 2.64 Hz. The LOD value was substituted into the regression equation *y* = 14.86*x* − 12.87 (R^2^ = 0.99), where x = log_10_(C), and the corresponding concentration was obtained as $C_{LOD}=10^{x_{LOD}}$. As a result, the calculated LOD is 11 ng mL^−1^. Since the LOD is close to the lowest experimentally tested MBP concentration (10 ng mL^−1^), this value should be interpreted within the investigated concentration range.

Although the responses at 5 and 10 µg mL^−1^ were not included in the regression analysis, they provide valuable information regarding the saturation behaviour of the sensor. At saturation, the averaged normalized frequency shift is approximately 52.3 ± 1.8 Hz. According to the Sauerbrey equation, this corresponds to an apparent MBP surface mass density of 360.7 ± 12.4 ng cm^−2^ and an estimated mass of 72.1 ± 2.5 ng. Since the estimated molecular weight of recombinant MBP is 21 kDa, the saturation response corresponds to an apparent population of 2.07 × 10^12^ MBP molecules on the sensing surface, which is close to the estimated aptamer population of 2.09 × 10^12^ molecules. The similar populations of MBP and aptamer suggest an approximate 1:1 binding stoichiometry at saturation. However, this estimation may also include hydrodynamically coupled water and counterions, as well as contributions from conformational rearrangements of aptamer molecules during binding.

**Figure 5:**
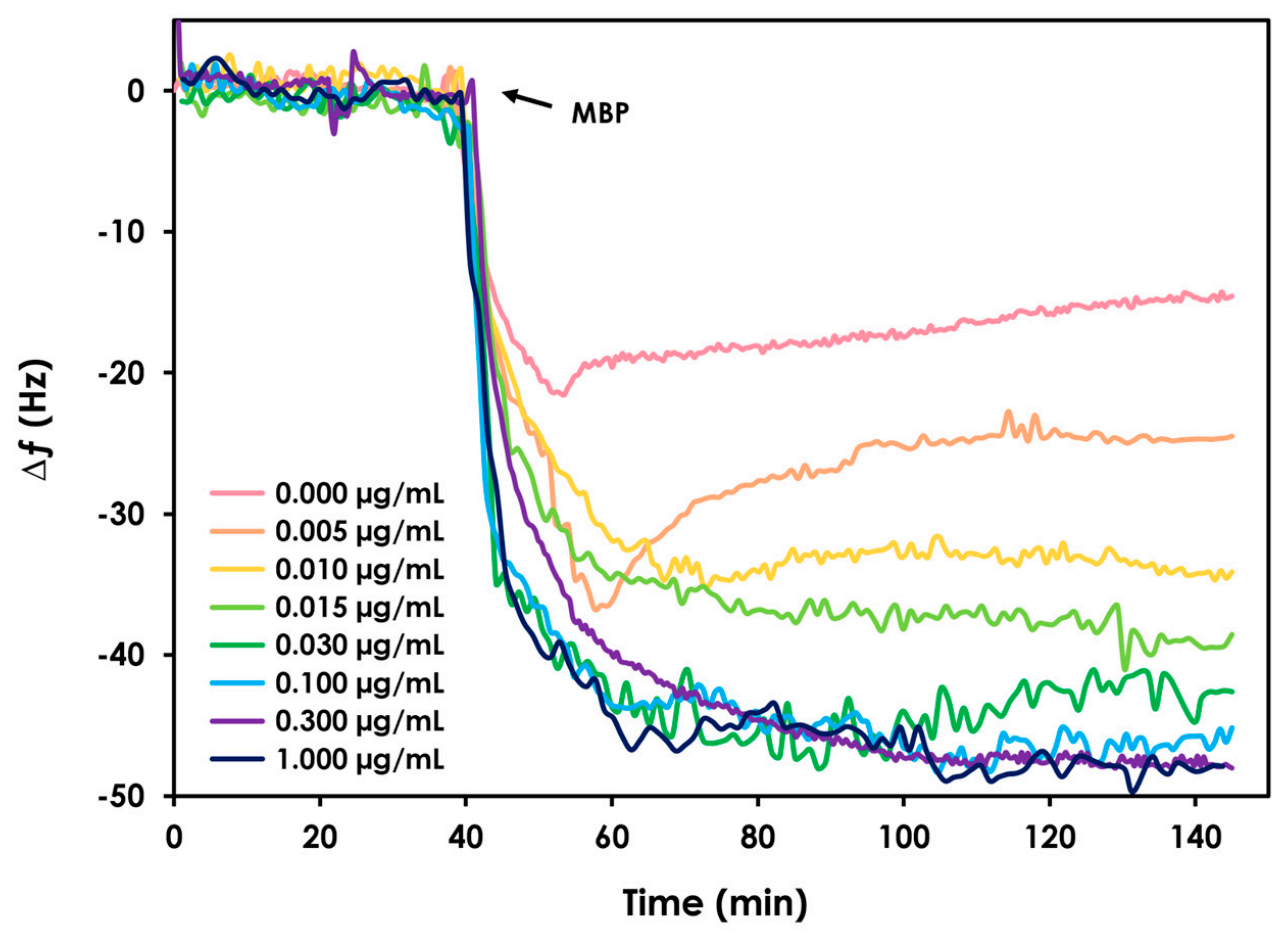
TSM sensor response to recombinant MBP in artificial cerebrospinal fluid. The arrow indicates addition of MBP to the aptasensor surface. The concentrations of MBP are shown in the inset.

**Figure 6:**
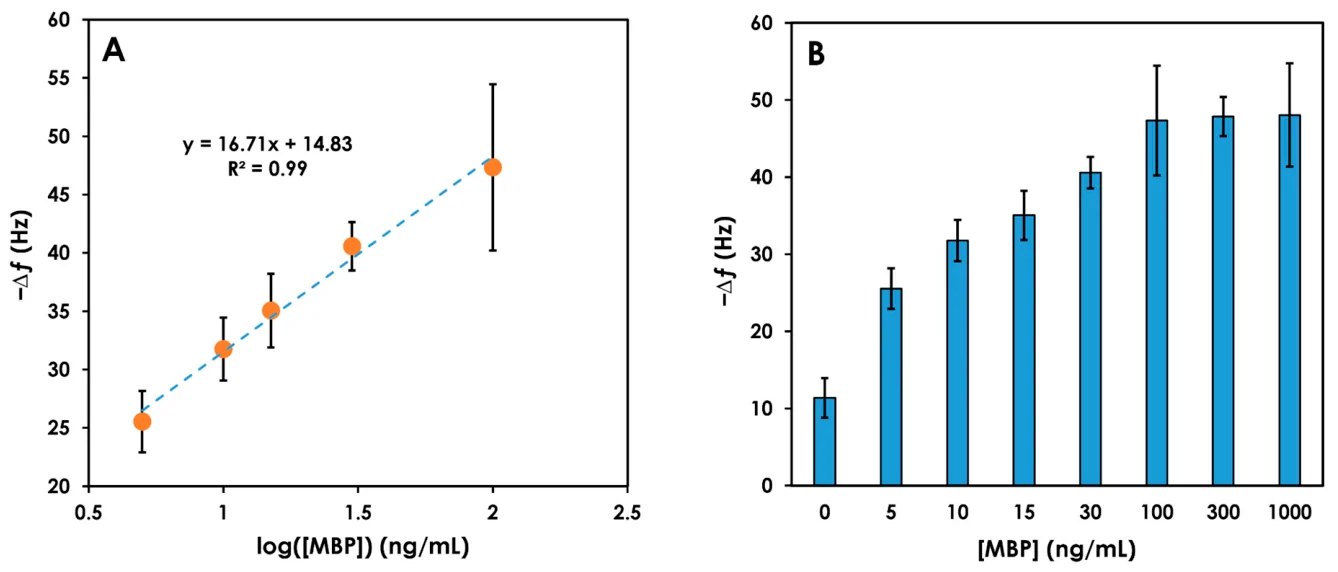
(**A**) The calibration curve plotting frequency shifts (∆ƒ) against the logarithm of recombinant MBP concentrations described by the linear regression equation, y = 16.71x + 14.83 (R^2^ = 0.99), and (**B**) average responses of the acoustic wave aptasensor to recombinant MBP in aCSF. The results are mean ± SD obtained from three independent experiments.

Detection of MBP in artificial cerebrospinal fluid resulted in a larger and more rapid signal change, indicating a matrix-associated contribution to the acoustic response (Figure 5). The signal also stabilized to a new baseline within one hour of exposing the disc to aCSF. Despite this matrix-associated response, increasing concentrations of MBP caused progressively greater decreases in frequency (Table S2). Importantly, aCSF contained 6.15 µM HSA, indicating that MBP detection was achieved in the presence of a relatively high concentration of albumin. Signal saturation occurred at a much lower concentration than in PBS, with similar results for all concentrations of MBP above 0.1 µg mL^−1^. A response was clearly observed at 0.005 µg mL^−1^ when measuring in aCSF. The corresponding calibration curve and average sensor response at various concentrations of MBP in aCSF is shown on the Figure 6.

To determine the LOD of the sensor in aCSF, a logarithmic scale of the MBP concentrations was used. The LODs in aCSF were calculated from the linear regression of the plots, according to the following equations:

$$LOB=mean\;blank\;+1.645\;{SD}_{blank}$$

$$LOD=LOB+1.645\;{SD}_{lc}$$

where the limit of blank (LOB) is the highest apparent concentration of MBP, ${SD}_{blank}$ is the standard deviation of the blank, the mean blank is the average of the blank, and ${SD}_{lc}$ is the standard deviation at the lowest concentration of the sample. 1.645 is the z-score, a statistical value derived from the normal distribution of blank values. By applying this value, an interval is considered that encompasses approximately 95% of the expected values for the blank.

To determine the concentration corresponding to the LOD, the LOD must be converted back to the concentration by applying the inverse of the regression equation and exponentiating the result:

$${LOD}_{concentration}=10^{(LOD-q)/m}$$

where *q* and *m* are the intercept and the slope of the linear regression, respectively.

For aCSF, the blank response was 11.4 ± 2.6 Hz (mean ± SD), and the standard deviation at the lowest tested MBP concentration (5 ng mL^−1^) was 2.6 Hz. Therefore, the calculated LOB was 15.68 Hz and the corresponding LOD signal was 19.95 Hz. Using the calibration equation y = 16.71x + 14.83 (R^2^ = 0.99), where x = log_10_([MBP] in ng mL^−1^), the calculated LOD corresponded to 2.0 ng mL^−1^.

The concentration-dependent response in aCSF was additionally analyzed with a Langmuir binding model [35] that incorporated a baseline offset to account for the response of the aCSF matrix in the absence of MBP:

$$-\Delta f\left(C\right)=\Delta f_{0}+\Delta f_{MAX}\frac{C}{K_{D}^{app}+C}$$

where $\Delta f_{0}$ represents the baseline response of the aCSF matrix in the absence of MBP, $\Delta f_{MAX}$ is the maximum MBP-associated frequency variation, *C* is the MBP concentration, and $K_{D}^{app}$ is the apparent dissociation constant. The data were fitted by weighted nonlinear least-squares regression using $\frac{1}{SD^{2}}$ as the weighting factor, where the SD was calculated from three independent measurements at each concentration. The fit produced a $K_{D}^{app}$ = 8.5 ng mL^−1^ and $\Delta f_{MAX}$ ≈ 37.5 Hz (R^2^ = 0.998). The fitted baseline, $\Delta f_{0}$ ≈ 11.4 Hz, was consistent with the experimentally measured response of aCSF without MBP (11.4 ± 2.6 Hz). Since $K_{D}^{app}$ was derived from the acoustic response upon aptamer immobilization, it should be considered as an apparent binding parameter under the experimental conditions rather than as an intrinsic solution-phase dissociation constant.

The PBS concentration-response data were also evaluated with the Langmuir model. However, the estimated $K_{D}^{app}$ showed greater dependence on the fitting approach than was observed in aCSF, with weighted and unweighted nonlinear regression producing values of approximately 269 and 442 ng mL^−1^, respectively. Due to this variability, a quantitative $K_{D}^{app}$ was not assigned for PBS.

The LODs in PBS and aCSF were calculated using different statistical approaches and over different experimental concentration ranges. Therefore, the LOD values should not be interpreted as a direct comparison of analytical sensitivity between the two experimental media. Furthermore, the PBS experiments primarily assessed the functionalization of the sensing surface and its functionality in terms of MBP recognition before evaluating the aptasensor in a complex matrix. As a result, a more extensive low-concentration range was investigated in aCSF, which is relevant to reported values of MBP in CSF samples.

The developed biosensor achieved an LOD of 2.0 ng mL^−1^ for MBP in aCSF. MBP concentrations in healthy individuals are typically below 4 ng mL^−1^, whereas elevated levels ranging from approximately 4–10 ng mL^−1^ and above 10 ng mL^−1^ have been reported in patients with demyelinating and other neurological disorders. Therefore, the calculated LOD falls below these reported concentration ranges. Importantly, determining the LOD in aCSF was mainly to evaluate whether MBP could be detected in a CSF model matrix at concentrations relevant to those reported in biological CSF samples. However, because these measurements were performed in aCSF rather than human CSF, validation in clinical samples is required to determine whether comparable analytical performance is maintained in biological specimens. The lower LOD and earlier saturation observed in aCSF should be interpreted in the context of the matrix-dependent acoustic response. Differences in ionic composition and matrix components may affect aptamer conformation, interfacial hydration, coupled water and ions, and nonspecific interactions, which can alter the magnitude and concentration dependence of the TSM-D response. The saturation response was also slightly lower in aCSF than in PBS (Δf_sat_ ≈ 47.8 Hz versus 52.3 Hz, respectively), further indicating matrix-dependent behaviour of the sensing interface. Furthermore, because the LODs in PBS and aCSF were calculated with different statistical procedures and experimental concentration ranges, the LODs are not directly comparable. Therefore, the lower LOD in aCSF should not be interpreted as improved affinity or analytical performance compared with PBS.

The analytical performance of the developed TSM-D aptasensor was compared with previously reported aptamer-based MBP biosensors (Table 1). Previous MBP aptasensors are based on electrochemical transduction and reported lower LODs than those obtained in the present study. However, in the present work, the TSM-D platform was applied to characterize the antifouling sensing surface through simultaneous monitoring of frequency and dissipation shifts under flow, providing information on sensor functionalization, MBP binding, and variations in the interfacial layer. The analytical range and saturation behaviour of the different MBP aptasensors are also summarized in Table 1 to provide a direct comparison of their analytical characteristics.

**Figure 7:**
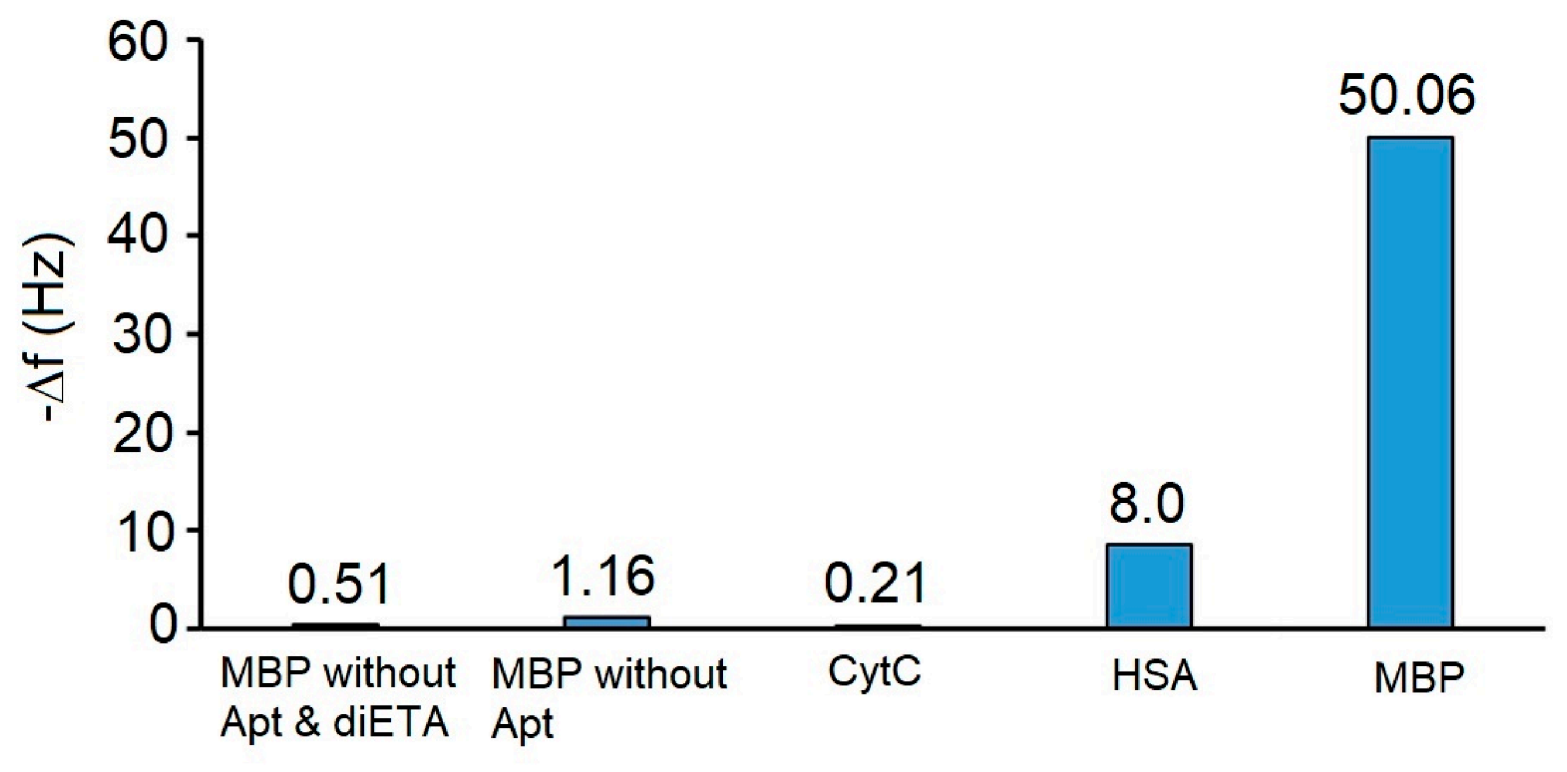
Selectivity analysis of the aptasensor for recombinant MBP. Cytochrome c (CytC) (10 μg mL^−1^) was chosen as a non-target cationic protein control to assess the contribution of nonspecific electrostatic interactions and human serum albumin (HSA) (6.15 μM) has been used to analyze the possible effect of the main protein component of aCSF. The concentration of MBP was 3 μg.mL^−1^.

The selectivity of the aptasensor was evaluated in PBS under different conditions (Figure 7). First, 3 µg mL^−1^ MBP was injected over the antifouling thiol SAM in the absence of aptamer and ethanolamine to assess nonspecific adsorption. Only a negligible frequency shift of −0.51 Hz was observed, indicating that minimal interaction between MBP and the thiol SAM layer occurred. Another experiment was performed in the absence of aptamer, while maintaining the blocking step with diethanolamine, and a frequency shift of −1.16 Hz was observed following incubation with MBP. The aptasensor surface was exposed also to 10 µg mL^−1^ cytochrome c (CytC), which was selected as a non-target control due to its relatively small size and strong positive charge at physiological pH, allowing the contribution of nonspecific electrostatic interactions to the sensor response to be assessed. CytC produced a frequency shift of only −0.21 Hz. The minimal response observed for CytC indicates that electrostatic attraction alone is insufficient to produce a measurable signal. We also examined the sensor response following incubation with 6.15 μM HSA, the main protein component of aCSF. In this case, the frequency shift was approximately −8 Hz. In contrast, introducing 3 µg mL^−1^ MBP to the aptasensor produced a greater frequency shift of −50.06 Hz. The MBP-associated response on the aptasensor surface was approximately 98-, 43-, 238 and 6-fold and greater than the frequency shifts obtained for the SAM-only, aptamer-free, CytC and HSA experiments, respectively. Therefore, the acoustic response during MBP measurements predominantly arises from selective recognition of recombinant MBP by the immobilized aptamer rather than nonspecific adsorption or electrostatic interactions. While the selectivity experiments involved aptamer-free surfaces and non-target protein controls, additional selectivity measurements suing a scrambled or non-cognate DNA sequence of comparable length represent an additional control that should be considered in future studies. Certain response of the sensor to the presence of HSA is comparable with the frequency shift in aCSF without MBP. Thus, the HSA is most probably the main contributor to the nonspecific response of the sensor in aCSF (Figure 6B).

Although LJM-5708 was derived from an aptamer originally selected against murine myelin, the present study demonstrates its binding to recombinant human MBP. As a consequence, the original study did not assess a dissociation constant for the interaction of the LJM-5708 aptamer with the murine MBP [13]. Therefore, a quantitative comparison with the $K_{D}^{app}$ calculated in this work for recombinant human MBP is not possible. Nevertheless, species differences, together with different isoforms and post-translational modifications of native human MBP, may affect aptamer recognition. Therefore, although recombinant human MBP provides a well-defined model for evaluating the sensing platform, future validation with native MBP in human CSF samples is required to determine its potential clinical applicability.

## 4. Conclusions

An acoustic wave aptasensor for the detection of recombinant myelin basic protein (MBP) was developed for label-free measurements in artificial cerebrospinal fluid (aCSF). The developed sensor used a DNA aptamer for MBP that was bound to an engineered antifouling mixed thiol layer on a piezoelectric TSM disc. Recombinant MBP was successfully expressed and characterized. At lower concentrations, MBP was predominantly non-aggregated, while concentrating MBP resulted in the formation of MBP aggregates. Detection of MBP was performed in phosphate-buffered saline (PBS) and aCSF. The limits of detection in PBS and aCSF were 11 ng mL^−1^ and 2 ng mL^−1^, respectively. Despite matrix-associated contributions to the acoustic response in aCSF, increasing MBP concentrations still produced measurable signals, with a lower limit of detection in aCSF than in PBS. Compared to bare gold, the aptasensor demonstrated significantly reduced frequency and dissipation responses upon exposure to aCSF due to the antifouling properties of the functionalized sensing surface. Furthermore, the selectivity experiments showed negligible responses for aptamer-free surfaces and upon exposure of the aptasensor to the non-target cationic protein cytochrome c, and lower response to the human serum albumin (HSA), supporting preferential recognition of recombinant human MBP. These results support the importance of the engineered antifouling interface for further development and evaluation of the aptasensor in real samples. Future surface-engineering strategies may further explore the roles of surface charge, hydrophilicity, hydration, and steric repulsion of the sensing interface. While aCSF provides a controlled matrix for evaluating sensor performance under more complex conditions than PBS, it does not reproduce the full biochemical complexity and biological variability of human CSF. Further validation using human CSF samples will therefore be required to establish sensor performance in clinically relevant specimens and assess its potential for disease monitoring.

## Figures and Tables

**Table 1:** Comparison of the analytical performance of previously reported aptamer-based MBP biosensors and the present TSM-D aptasensor; NR: not reported.

Target	Transduction	Matrix	LOD (ng mL^−1^)	Analytical Range (ng mL^−1^)	Saturation (ng mL^−1^)	Reference
Mouse MBP	EIS	aCSF	0.01	0.01–64	NR	[14]
Human MBP	EIS	aCSF	0.65	1–128	NR	[15]
Human MBP	EIS	Human serum	0.35	0.35–128	NR	[36]
Recombinant human MBP	TSM-D	PBS	11	10–3000	5000	This work
Recombinant human MBP	TSM-D	aCSF	2.0	5–100	300	This work

## Data Availability

The data will be available upon request.

## Supplementary Materials

The following supporting information can be downloaded at https://www.mdpi.com/article/10.3390/bios16100564/s1. Figure S1: The expression plasmid pRSET_B for recombinant MBP. Figure S2: SDS-PAGE with the following lanes: (A) the protein ladder, (B) column flow-through, (C) eluted recombinant MBP at approximately 21 kDa, and (D–F) His-binding buffer washes. Figure S3: The UV-Vis absorbance of purified recombinant MBP. Figure S4: Dynamic light scattering of purified recombinant MBP in HEPES buffer: (A) number distribution shows a main protein population with a hydrodynamic diameter of 9.12 ± 1.46 nm; (B) intensity distribution includes minor populations of MBP aggregates; (C) volume distribution confirms that 99.3% of the sample is made up of a protein population with a hydrodynamic diameter of approximately 9.53 ± 1.54 nm, indicating that trace amounts of aggregates are present. Figure S5: Particle size distributions of concentrated recombinant MBP. Progressive concentration-dependent self-association and aggregation of recombinant His-tagged myelin basic protein (MBP). Representative dynamic light scattering (DLS) particle size distributions of concentrated MBP preparations obtained following centrifugal spin filtration. Number-weighted (left column) and intensity-weighted (right column) distributions are shown for Sample 1 (A, B), Sample 2 (C, D), and Sample 3 (E, F). The number-weighted distributions demonstrate a progressive increase in the predominant particle diameter from approximately 50 nm to 98 nm and 175 nm, while the corresponding intensity-weighted distributions reveal the appearance of increasingly larger aggregates, illustrating the concentration-dependent self-association behaviour of recombinant MBP. Figure S6: Affinity binding of recombinant His-tagged MBP to a Ni-NTA-functionalized TSM disc. Figure S7: Comparison of the TSM-D response of bare gold and the complete aptasensor upon exposure to aCSF: (A) frequency response and (B) dissipation response. Table S1. Average TSM-D frequency response to recombinant MBP in phosphate-buffered saline. Table S2. Average TSM-D frequency response to recombinant MBP in artificial cerebrospinal fluid.

## Abbreviations

The following abbreviations are used in this manuscript:

ab-NTA	N(*α*), N(*α*) bis-(carboxymethyl)-L-lysine disodium salt monohydrate
aCSF	Artificial Cerebrospinal Fluid
ADEM	Acute Disseminated Encephalomyelitis
CFT	Column Flow-Through
citMBP	Citrullinated Myelin Basic Protein
CMT	Charcot-Marie-Tooth disease
CNS	Central Nervous System
CSF	Cerebrospinal Fluid
CytC	Cytochrome c
DLS	Dynamic Light Scattering
DTT	Dithiothreitol
EDC	N-(3-dimethylaminopropyl)-N’-ethyl carbodiimide hydrochloride
EIS	Electrochemical Impedance Spectroscopy
ELISA	Enzyme-Linked Immunosorbent Assay
GBS	Guillain-Barré Syndrome
HSA	Human serum albumin
HS-MEG-COOH	3-(2-mercaptoethoxy)propanoic acid
HS-MEG-OH	2-(2-mercaptoethoxy)ethanol
HS-MIX	mixed self-assembled monolayer
IPTG	isopropyl *β*-D-1-thiogalactopyranoside
LB	Lysogeny Broth
LOB	Limit of Blank
LOD	Limit of Detection
MBP	Myelin Basic Protein
MS	Multiple Sclerosis
NHS	N-hydroxysuccinimide
PBS	Phosphate-buffered Saline
PNS	Peripheral Nervous System
SDS-PAGE	SDS polyacrylamide gel electrophoresis
SELEX	Systematic Evolution of Ligands by Exponential Enrichment
TSM-D	Thickness Shear Mode with Dissipation
