# Supplementary material for: Label-Free Acoustic Wave Detection of Myelin Basic Protein for Monitoring of Neurodegenerative Disease
Source: Biosensors (Basel). 2026 Oct 8;16(10):564. doi: 10.3390/bios16100564 (PMC13702401; doi:10.3390/bios16100564)
Supplement: Supplementary file 1 [file biosensors-16-00564-s001.zip › biosensors-4537040-supplementary.pdf]

Supplementary Materials

Label-Free Acoustic Wave Detection of Myelin Basic Protein for Monitoring Neurodegenerative Disease

Sandro Spagnolo[1,*], Sára Mikuličová[1], Katharina Davoudian[2], Kiran Sontakke[1], Brian De La Franier[2], Michael Thompson[2,*], and Tibor Hianik[1]

[1] Faculty of Mathematics, Physics and Informatics, Comenius University, Mlynská dolina F1, 84248 Bratislava, Slovakia; sara.mikulicova@fmph.uniba.sk (S.M.); kiran.sontakke@fmph.uniba.sk (K.S.); tibor.hianik@fmph.uniba.sk (T.H.)

[2] Department of Chemistry, University of Toronto, 80 St. George Street, Toronto, ON M5S 3H6, Canada; k.davoudian@mail.utoronto.ca (K.D.); brian.delafranier@mail.utoronto.ca (B.D.L.F.)

* Correspondence: spagnolo2@uniba.sk (S.S.) and m.thompson@utoronto.ca (M.T.)

S1. The expression plasmid pRSET_B.

The amino acid sequence of recombinant MBP is the following: MASQKRPSQR HGSKYLATAS TMDHARHGFL PRHRDTGILD SIGRFFGGDR GAPKRGSGKV PWLKPGRSPL PSHARSQPGL CNMYKDSHHP ARTAHYGSLP QKSHGRTQDE NPVVHFFKNI VTPRTPPPSQ GKGRGLSLSR FSWGAEGQRP GFGYGGRASD YKSAHKGFKG VDAQGTLSKI FKLGGRDSRS GSPMARR.

Figure S1 is a schematic map of the pRSET_B plasmid used for the expression of His-tagged MBP in *Escherichia coli* BL21 (DE3). The construct contains the T7 promoter, Xpress™ epitope, 6×His tag, MBP coding sequence, and the *bla* gene to provide ampicillin resistance.

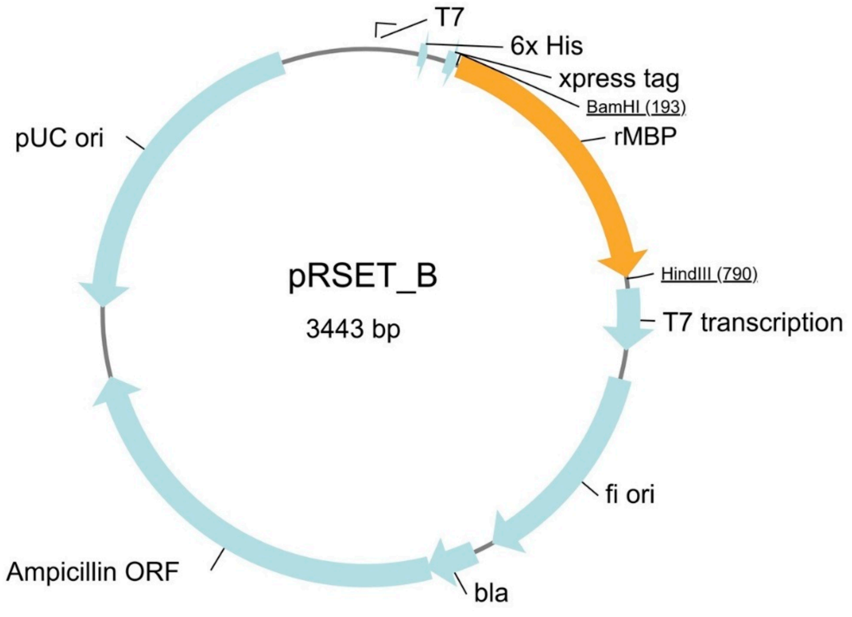

Figure S1. The expression plasmid pRSET_B for recombinant MBP.

S2. Characterization of the purification fractions by SDS-PAGE

SDS-PAGE analysis was performed to evaluate the expression and purification of recombinant MBP (Figure S2). The gel shows the composition of the column flow-through, elution, and wash fractions during nickel-NTA affinity purification.

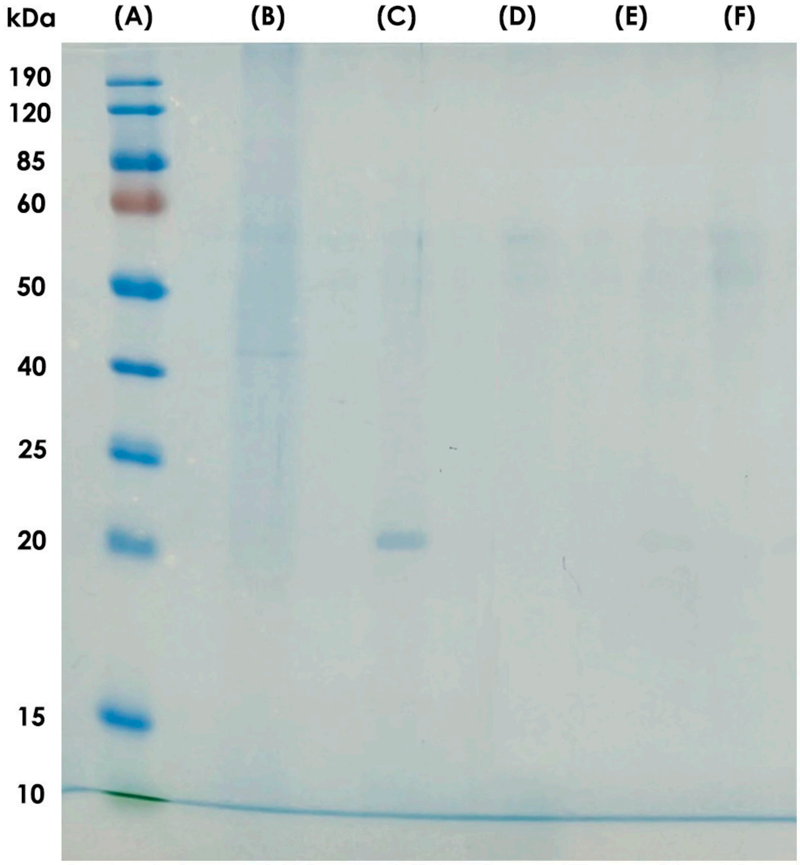

Figure S2. SDS-PAGE with the following lanes: (A) the protein ladder, (B) column flow-through, (C) eluted recombinant MBP at approximately 21 kDa, and (D–F) His-binding buffer washes.

S3. UV–Vis Spectroscopic Characterization of Recombinant MBP

UV-Vis absorption spectroscopy was performed to calculate the concentration of recombinant MBP (**Figure S3**). The spectrum exhibited an absorbance at 276 nm, which was used to estimate the protein concentration according to the Beer–Lambert law.

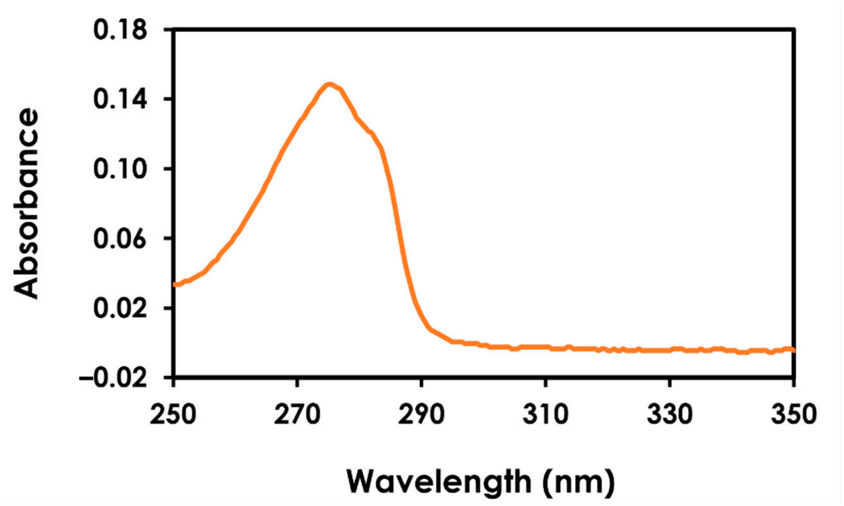

Figure S3. The UV-Vis absorbance of purified recombinant MBP.

S4. Dynamic Light Scattering Analysis of Purified Recombinant MBP

Dynamic light scattering (DLS) was performed to characterize the hydrodynamic size distribution of purified recombinant MBP at an initial protein concentration of 0.157 mg mL^{-1} (**Figure S4**).

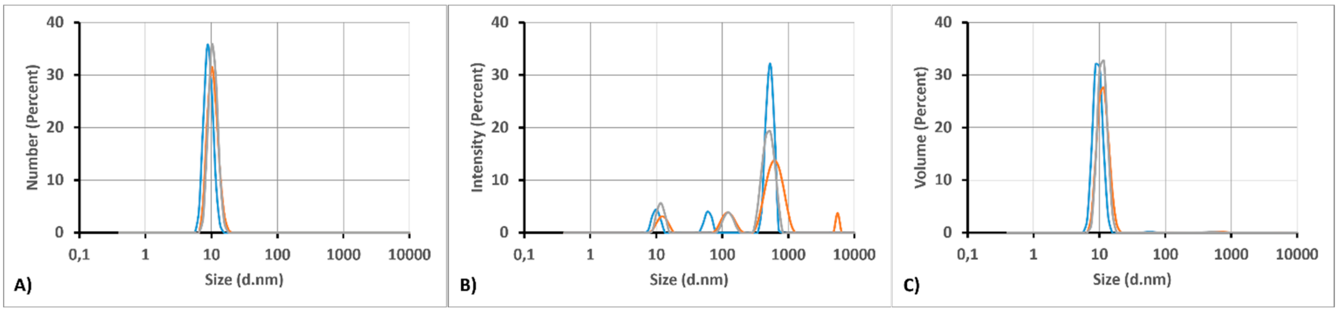

Figure S4. Dynamic light scattering of purified recombinant MBP in HEPES buffer: (A) number distribution shows a main protein population with a hydrodynamic diameter of 9.12 ± 1.46 nm, (B) intensity distribution includes minor populations of MBP aggregates, (C) volume distribution confirms that 99.3% of the sample is made up of a protein population with a hydrodynamic diameter of approximately 9.53 ± 1.54 nm, indicating that trace amounts of aggregates are present.

S5. Concentration-Dependent Aggregation of Recombinant MBP

The initial protein suspension of recombinant MBP was progressively concentrated by centrifugal spin filtration and analysed by DLS to evaluate the effect of protein concentration on the hydrodynamic behaviour of MBP (**Figure S5**).

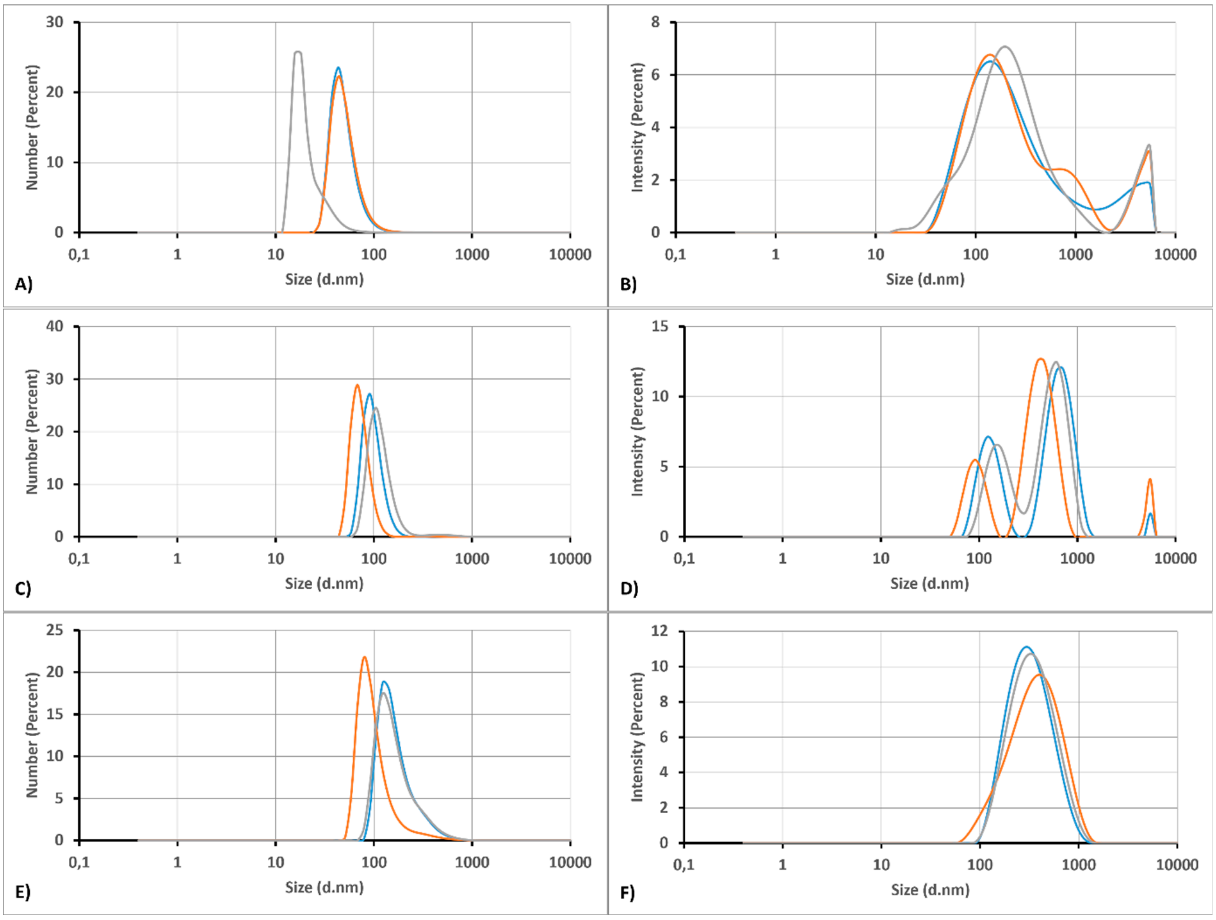

Figure S5. Particle size distributions of concentrated recombinant MBP. Progressive concentration-dependent self-association and aggregation of recombinant His-tagged myelin basic protein (MBP). Representative dynamic light scattering (DLS) particle size distributions of concentrated MBP preparations obtained following centrifugal spin filtration. Number-weighted (left column) and intensity-weighted (right column) distributions are shown for Sample 1 (A, B), Sample 2 (C, D), and Sample 3 (E, F). The number-weighted distributions demonstrate a progressive increase in the predominant particle diameter from approximately 50 nm to 98 nm and 175 nm, while the corresponding intensity-weighted distributions reveal the appearance of increasingly larger aggregates, illustrating the concentration-dependent self-association behaviour of recombinant MBP.

S6. Confirmation of Recombinant MBP Binding to Ni–NTA-Functionalized TSM Discs

A representative frequency response was observed during the affinity binding of recombinant MBP to a Ni-NTA-functionalized TSM disc, followed by washing with PBS (**Figure S6**). Measurements were conducted at the third overtone, and the frequencies shown in the graph are not normalized.

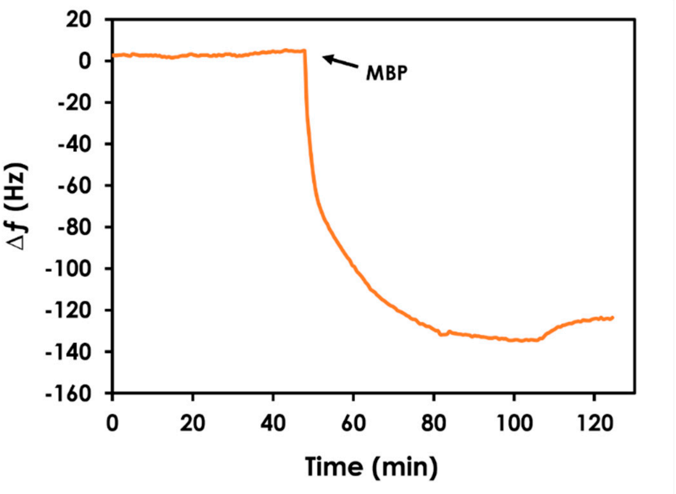

Figure S6. Affinity binding of recombinant His-tagged MBP to a Ni-NTA-functionalized TSM disc.

S7. Antifouling Performance of the Sensing Interface in aCSF

The antifouling performance of the HS-MEG-COOH-based sensing interface was evaluated by comparing the TSM-D response of bare gold (in blue) and the complete aptasensor (in orange) upon exposure to aCSF. Representative frequency and dissipation responses are shown in **Figure S7**.

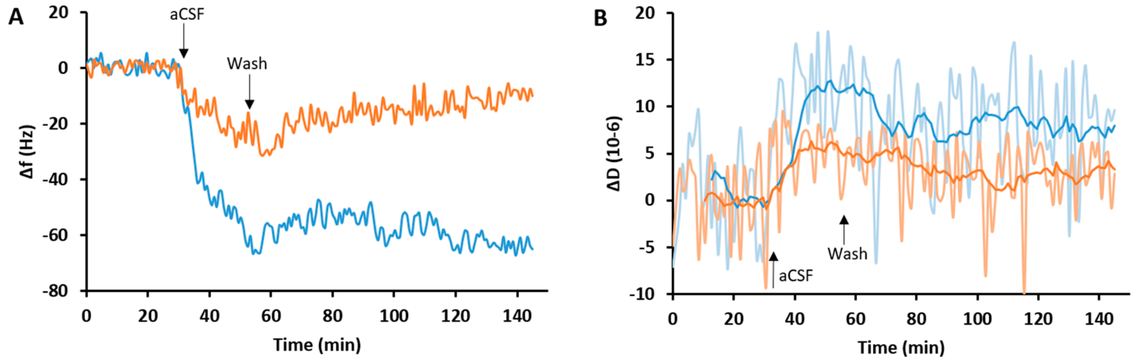

Figure S7. Comparison of the TSM-D response of bare gold and the complete aptasensor upon exposure to aCSF: (A) frequency response and (B) dissipation response.

S8. Detection of Recombinant MBP in PBS

Table S1. Average TSM-D frequency response to recombinant MBP in phosphate-buffered saline.

MBP Concentration (µg mL^{-1})	$-\Delta f$ (Hz)	Standard Deviation (Hz)
**0.01**	3.3	0.8
**0.10**	16.3	1.3
**0.30**	22.3	2.4
**1.00**	30.5	4.9
**3.00**	41.1	3.1
**5.00**	51.7	1.5
**10.0**	52.9	2.2

S9. Detection of Recombinant MBP in aCSF

Table S2. Average TSM-D frequency response to recombinant MBP in artificial cerebrospinal fluid.

MBP Concentration (μg mL^{-1})	$-\Delta f$ (Hz)	Standard Deviation (Hz)
**0.000**	11.4	2.6
**0.005**	25.5	2.6
**0.010**	31.8	2.7
**0.015**	35.1	3.2
**0.030**	40.6	2.1
**0.100**	47.4	7.1
**0.300**	47.9	2.5
**1.000**	48.0	6.7
